# Remotely Triggered Scaffolds for Controlled Release of Pharmaceuticals

**DOI:** 10.3390/ijms14048585

**Published:** 2013-04-19

**Authors:** Paul Roach, David J. McGarvey, Martin R. Lees, Clare Hoskins

**Affiliations:** 1 Guy Hilton Research Centre, Institute of Science and Technology in Medicine, Keele University, Keele, ST4 7QB, UK; E-Mail: p.roach@keele.ac.uk; 2 Lennard-Jones Laboratories, School of Physical and Geographical Sciences, Keele University, Keele, ST5 5BG, UK; E-Mail: d.j.mcgarvey@keele.ac.uk; 3 Physics Department, University of Warwick, Coventry, CV4 7AL, UK; E-Mail: m.r.lees@warwick.ac.uk; 4 School of Pharmacy, Institute of Science and Technology in Medicine, Keele University, Keele, ST5 5BG, UK

**Keywords:** scaffold, smart material, thermo-responsive, hybrid nanoparticle, surface plasmon

## Abstract

Fe_3_O_4_-Au hybrid nanoparticles (HNPs) have shown increasing potential for biomedical applications such as image guided stimuli responsive drug delivery. Incorporation of the unique properties of HNPs into thermally responsive scaffolds holds great potential for future biomedical applications. Here we successfully fabricated smart scaffolds based on thermo-responsive poly(*N*-isopropylacrylamide) (pNiPAM). Nanoparticles providing localized trigger of heating when irradiated with a short laser burst were found to give rise to remote control of bulk polymer shrinkage. Gold-coated iron oxide nanoparticles were synthesized using wet chemical precipitation methods followed by electrochemical coating. After subsequent functionalization of particles with allyl methyl sulfide, mercaptodecane, cysteamine and poly(ethylene glycol) thiol to enhance stability, detailed biological safety was determined using live/dead staining and cell membrane integrity studies through lactate dehydrogenase (LDH) quantification. The PEG coated HNPs did not show significant cytotoxic effect or adverse cellular response on exposure to 7F2 cells (*p* < 0.05) and were carried forward for scaffold incorporation. The pNiPAM-HNP composite scaffolds were investigated for their potential as thermally triggered systems using a Q-switched Nd:YAG laser. These studies show that incorporation of HNPs resulted in scaffold deformation after very short irradiation times (seconds) due to internal structural heating. Our data highlights the potential of these hybrid-scaffold constructs for exploitation in drug delivery, using methylene blue as a model drug being released during remote structural change of the scaffold.

## 1. Introduction

Advancement in knowledge surrounding cell-material interactions has led to development of novel materials for use in implant technology [1], *in vitro* expansion of cells for regenerative medicine [2] and medical diagnostics [3]. Scaffolds fabricated for tissue engineering purposes are required to be of controlled structure [4] and pore size [5] in order to allow diffusion of nutrients and toxins to in-growing tissue [6]. Cells require a suitable environment in order to develop into a 3D structure. Scaffold materials provide the architecture to support 3D tissue growth, although often such materials cause problems for long-term regeneration [7]. Biodegradable scaffolds that collapse after a fixed time period, either naturally or after external stimulus, are advantageous for long term use, e.g., to support tissue development or act as slow drug release carriers [8]. Addition of nanoparticles into the intrinsic structure of a scaffold can enhance functionality and lead to exciting new properties such as ability to remotely trigger structural collapse which could ultimately lead to drug release. Gold coated iron oxide hybrid nanoparticles (HNPs) have shown increasing potential for biomedical applications [9–11]. Fabrication of these hybrids can be carried out with ease producing particles of uniform size and physicochemical properties [10]. The iron oxide core can be used for external guidance [8,12] or as a contrast agent in magnetic resonance imaging (MRI) [13]. The rigid gold coat possesses surface plasmon characteristics [14]. This phenomenon results in the rapid conversion of light to heat energy upon specific irradiation of the particles [15]. It is desirable to produce particles with optimal absorption wavelengths within the “biological window” were light is highly transmitted through tissues but can offer a means of remote thermally switching [14]. This range is between 750 and 850 nm [15]. Incorporation of these nanoparticles into a biocompatible scaffold, results in a more sophisticated system with increased functionality for tissue engineering. Here, the heating of particles due to laser irradiation can be exploited in order to deform the three dimensional structure of the scaffold. Ultimately, growth factors or drug molecules could be incorporated onto the scaffold pores which can be thermally released into the surrounding tissues. Fabrication of such a system would result in the capability of focused release of bioactive molecules and a thermally degrading scaffold for tissue engineering. Additionally, the dense nanoparticle cores will also provide contrast in MRI imaging which can be used to visualize and monitor scaffold position and degradation.

Poly(*N*-isopropylacrylamide) (pNiPAM) is a thermally manipulated polymer which changes conformation in a reversible coil to globule manner at its lower critical solution temperature (LCST) [16]. Below the LCST pNiPAM exposes hydrophilic segments to the surrounding aqueous environment promoting swelling, however, when the LCST is exceeded the polymer forms a compact precipitate in the aqueous environment [17]. The LCST for pNiPAM is approximately 32 °C [18]. This conformational change has been exploited for drug delivery in a host of vehicles whereby, drugs trapped in the swollen polymer conformation exhibit rapid release as temperature increases past the LCST. Examples of such systems include amphiphilic copolymer micelles [19,20], coatings on inorganic nanoparticles [21,22] and medical devices such as stents [23,24]. Studies have reported thermal manipulation of iron oxide-pNiPAM [25] and gold nano-rod-pNiPAM [26] composites through induced hyperthermia in alternating magnetic fields and laser irradiation respectively. However, to the best of our knowledge the use of multimodal HNP-pNiPAM scaffold composites thermally triggered using laser irradiation has not yet been reported.

In this study hybrid iron oxide-gold nanoparticles were synthesized and characterized in terms of their bioactivity on mouse osteoclast (7F2) cells. Potential as nano-heaters via laser ablation was also characterized using an Nd:YAG laser whilst monitoring global heating effects of the bulk scaffold. HNPs were incorporated into the intrinsic structure of pNiPAM based scaffolds. The effect of laser ablation on the structural deformation and temperature change was observed, with demonstration of controlled drug release being shown using methylene blue to give an appreciation of the potential of such systems for triggered release of pharmaceuticals.

## 2. Results and Discussion

### 2.1. NiPAM Scaffold Charcteristics

**Figure 1:**
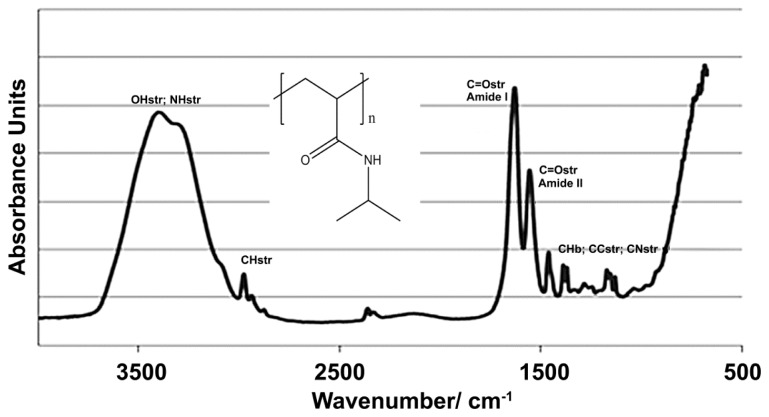
Representative FTIR spectra of pNiPAM polymer used in scaffold formation, with insert showing chemical structure.

**Figure 2:**
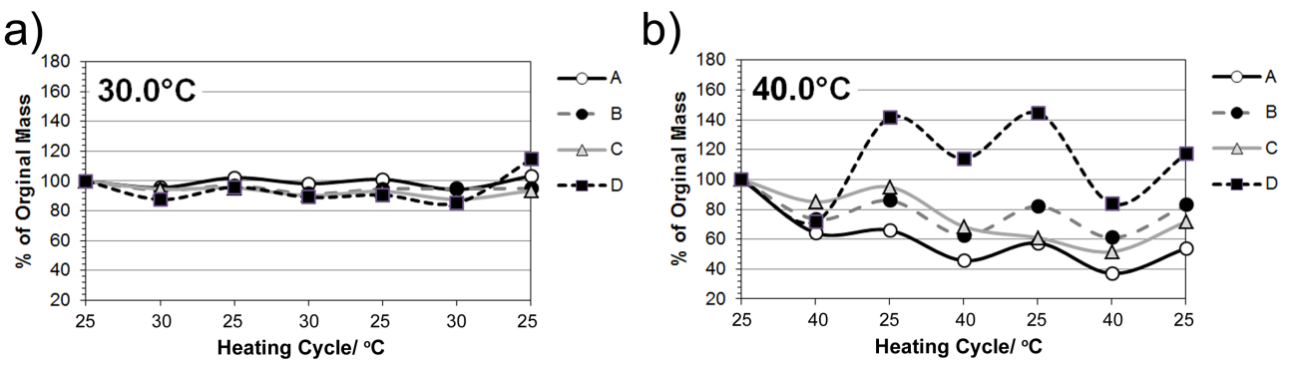

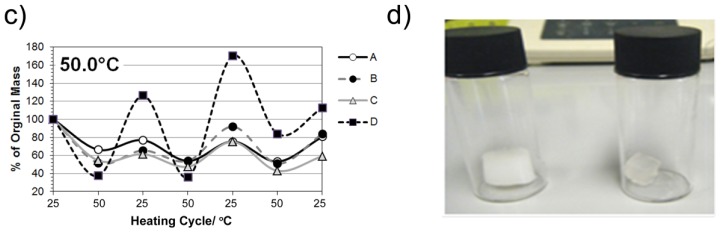
Cyclic heating properties of pNiPAM gels at (**a**) 30 °C; (**b**) 40 °C and (**c**) 50 °C. (**d**) Image of gels remaining shrunk when solvent removed after heating and returned to room temperature.

Scaffolds were produced via polymerization of *N*-Isopropylacrylamide to form a thermo-responsive pNiPAM network. The FTIR spectra for pNiPAM (Figure 1) shows expected bands at 3500–3200 cm^−1^, 1600 and 1500 cm^−1^ and 2800 and 1400 cm^−1^ arising from the N–H, C=O and C–H bonding respectively, matching well to expected structure of the polymer. pNiPAM polymers possess a unique ability, when exposed to changes in temperature, whereby interaction between polymer chains and aqueous solvent promotes collapse of the structure above the LCST, hence deforming bulk morphology. Here, the scaffolds were produced as thin surface coatings (~0.25 mm thick) on glass coverslips for cell culture or as monoliths for laser deformation and drug release studies. A range of gels were produced having differences in chain length and degree of cross-linking, afforded by change in ratio of NiPAM monomer and MBA cross-linker (Table 1). Gel elasticity was investigated in order that gels having optimal control over thermal switch deformation, whilst retaining the ability to undergo repeated switching cycles, were formed. Swelling properties were examined via immersion into temperature controlled water bath set at 25 °C, 30 °C, 40 °C and 50 °C (returning to room temperature between each cycle). The degree of remaining mass following solvent expulsion during shrinking was assessed in terms of repeated heating cycles. Successive heating and cooling cycles were found to impact on gel characteristics as shown in Figure 2. All gels were initially found to reduce in mass after expulsion of solvent when heated, with higher temperatures giving rise to larger degrees of shrinkage for all gels tested. At higher temperatures expansion was observed in one of the gels above its original cast volume. At 30 °C small changes up to ~15% of initial mass were recorded for all scaffolds, with a higher degree of cross-linking being found to restrict this change as would be expected. Gels showing the largest change, especially apparent in their expansion after heating to 50 °C were those with shorter chain length and a lower degree of cross-linking. These gel systems can be used for contractile and expansion of a scaffold structure via externally applied heating (and cooling) [27]. Repeated cycles showed that the scaffolds were elastically deforming under these conditions and so would be useful for repeated expulsion of solvent (incorporating drug molecules). Gels could be reduced in volume by heating and remained at this volume if solvent was removed, even upon cooling to 4 °C, Figure 2D. When re-solvated these gels slowly expanded back to their original mass over several minutes.

### 2.2. Synthesis and Characterization of Hybrid Nanoparticles

**Figure 3:**
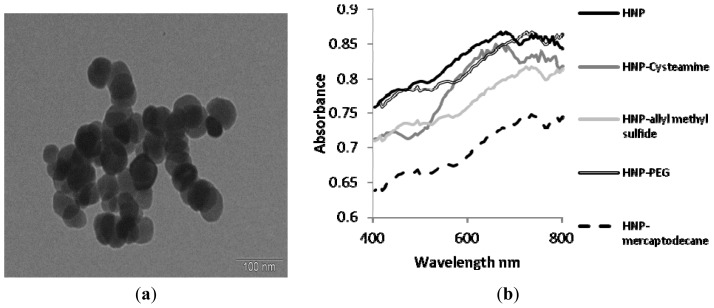
Characterization of hybrid nano-particles. (**a**) Transmission electron microscopy image of nano-particles and (**b**) UV-Vis spectroscopy of nano-particles suspended in deionized water.

**Figure 4:**
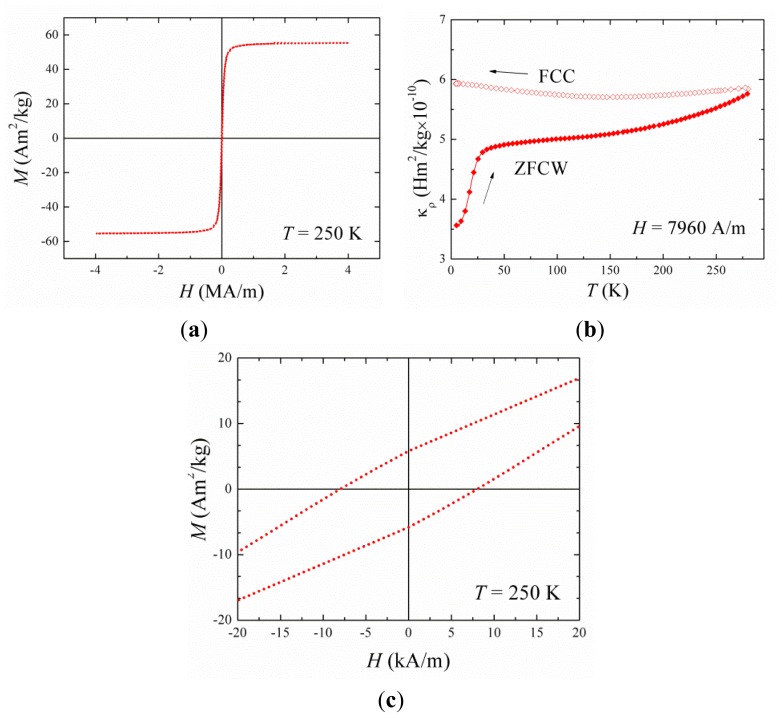
Magnetization data of Fe_3_O_4_-Au hybrid nanoparticles (HNPs). (**a**) M(T) curves measured in zero-field cooled warming and field-cooling mode; (**b**) M(H) curves collected at 250 K between −4 and 4 MA/m and (**c**) M(H) curves measured at 250 K.

The synthesis and characterization of hybrid nanoparticles was carried out via precipitation of iron oxide with subsequent electrochemical gold coating. A polymer intermediate was sandwiched between the iron oxide core and gold coating to protect the inherent properties of the constituent components. Gold coating was achieved by seeding followed by iterative reduction of acidic gold onto the nanoparticle surface. The gold coating step was closely monitored using zeta potential measurement of the particles in water and the metal content was determined using inductively coupled plasma–optical emission spectroscopy (ICP-OES) (Table 2). As gold coating was achieved the zeta potential dropped from the initial +47.4 mV of the PEI coated iron oxide to +10.5 mV, this was due to the electronegativity of the gold seeds and ultimately the coat reducing the overall surface charge. Particle diameter appeared to decrease over the coating steps; this can be attributed to the rigid gold coating forming around the flexible PEI hence, reducing its hydrodynamic diameter and minimizing aggregation due to the particles inherent magnetism (Table 2). Figure 3a shows the TEM image of the gold coated nanoparticles formed with average particle diameter of 70 nm (*n* = 20, measured using ANALYsis software). The images also indicate that the gold coating was achieved on individual iron oxide nanoparticles and not on particle clusters. UV spectroscopy of the hybrid nanoparticles showed peak absorbance at 690 nm (Figure 3b). This wavelength is indicative of the plasmon resonance of the gold shell. The presence of the peak also indicates that the gold coating was successful as without complete coating the individual gold seeds resonate at around 520 nm. Figure 4a shows the magnetization *vs.* applied field hysteresis loop collected at 250 K for the samples. The magnetic coercivity and saturation magnetization values at *T* = 250 K are indicative of highly crystalline Fe_3_O_4_ nanoparticles which are magnetically ordered at room temperature. The zero field cooled/field cooled curve (Figure 4b) shows features typical of ferromagnetic nanoparticle. The absence of a maximum in this data shows that the ordering temperature is above room temperature.

**Figure 5:**
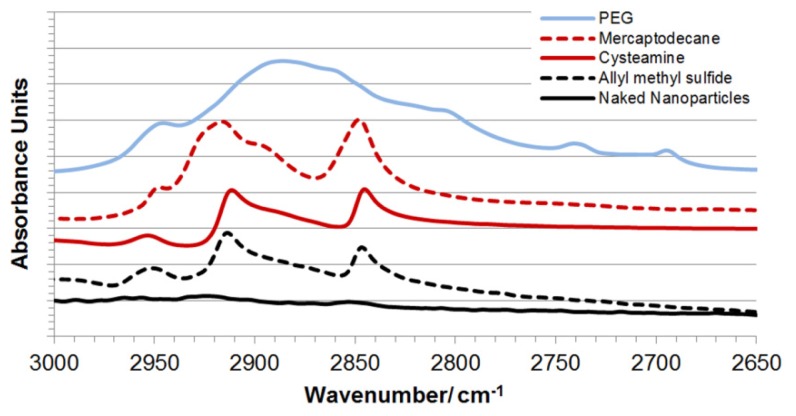
DRIFT FTIR spectra of functionalised hybrid nanoparticles. CH stretch region indicating presence of linker-surface groups.

In order for these iron oxide-gold coated nanoparticles to become biologically applicable they first require surface engineering with bioactive or biocompatible molecules, polymers or ligands. Hence, the nanoparticles were further functionalized through dative covalent bonding with four thiol or sulfide containing entities including cysteamine, allyl methyl sulphide, poly(ethylene glycol) thiol (PEG-Thiol) and mercaptodecane. The coated nanoparticles were characterized using ICP-OES, zeta potential measurement, photon correlation spectroscopy (Table 2), UV-Vis spectroscopy (Figure 3b) and DRIFT FTIR (Figure 5). FTIR spectra show the presence of aliphatic CH stretching bands associated with the presence of the functional group backbones. After coating the UV peak absorption was further red shifted towards the NIR region (approx. 720 nm). Thus, for optimal thermal effect laser irradiation should be carried out at this wavelength. The coating process for all HNPs was successful, which on visual inspection, could be observed by their greater solution stability compared with their uncoated counterparts.

### 2.3. Biological Characterization of Polymer Coated Hybrid Nanoparticles

**Figure 6:**
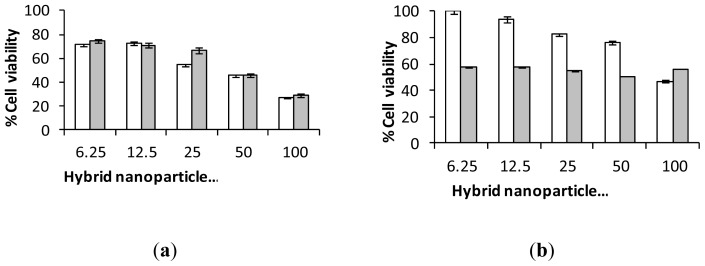

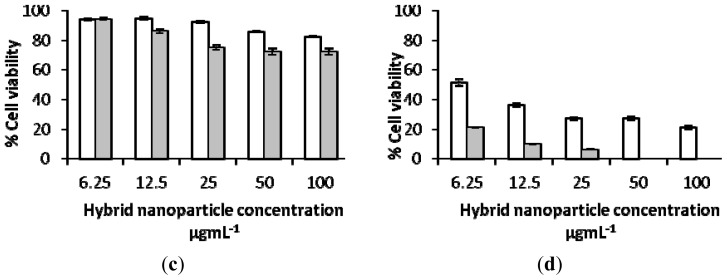
Cytotoxicity of polymer coated nano-hybrids on 7F2 cells over □ 24 h and ■ 72 h. Coating with (**a**) cysteamine; (**b**) allyl methyl sulphide; (**c**) PEG and (**d**) mercaptodecane.

**Figure 7:**
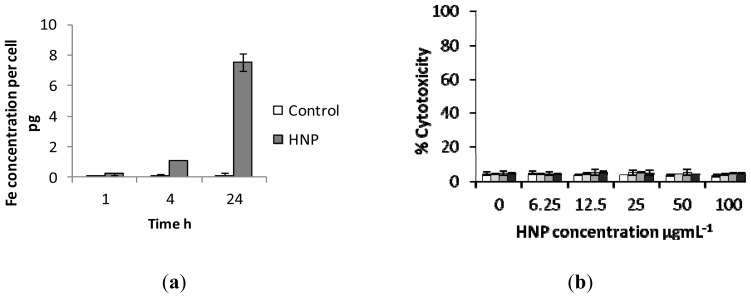
Biological investigations of nano-particle exposure in 7F2 cells. (**a**) Cellular uptake of HNP-PEG into 7F2 cells analysed using ICP-OES and (**b**) cell membrane integrity assay measured via lactate dehydrogenase quantification.

Cytotoxicity studies were carried out via trypan blue exclusion using mouse osteoblast (7F2) cells (Figure 6a–d). The results showed that the PEGylated nanoparticles (HNP-PEG) possessed the lowest cytotoxicity on 7F2 cells over the study period (72 h) with over 80% cell viability observed at the highest incubation concentration (100 μg mL^−1^). Whereas, after 72 h at 100 μg mL^−1^, only 40% and 60% viability remained in the cells incubated with the HNP-cysteamine and HNP-allyl methylsulfide respectively. No viable cells were observed after exposure to HNP-mercaptodecane at this concentration after the study period. These findings are not surprising given the wealth of literature on the biocompatible properties of PEG [28–31]. PEG is an FDA approved polymer routinely used for drug transfection [32], delivery [33] and in medical device coatings [34]. Hence, for all subsequent experiments the PEG coated nanoparticles were employed. Cellular internalization studies showed that up to 1 pg of HNP-PEG was taken up into the cells after only 4 h which increased to 7 pg after 24 h (Figure 7a). This indicated that cellular uptake occurred in a time dependent manner. The effect of nanoparticle exposure on cell membrane integrity was analyzed via lactate dehydrogenase (LDH) quantification (Figure 7b). Here an increase in LDH levels would be experienced if any disruption of the cell membrane had occurred as a result of nanoparticle exposure. The data suggests that no notable membrane damage had occurred as no excess LDH was observed compared with the basal levels in the 7F2 control cells. These biological studies indicate that the HNP-PEG did not trigger any adverse biological response *in vitro* on this cell line. Previous work has also shown that the HNP-PEG does not result in an increase in cellular stress levels due to free radical generation through reactive oxygen species and lipid peroxidation on human pancreatic adenocarcinoma (BxPC-3) and differentiated human monocyte (U937) cells [35]. Taking into account our new data combined with previous toxicological studies on HNPs [35], there is no observable reason why the HNPs would not be appropriate for incorporation into the integral pNiPAM scaffold structure. Incorporation into the pNiPAM structure results in the potential for laser manipulation of the scaffold which could be used for stimuli responsive drug release.

### 2.4. Laser Irradiation of HNP-PEG Phantoms

**Figure 8:**
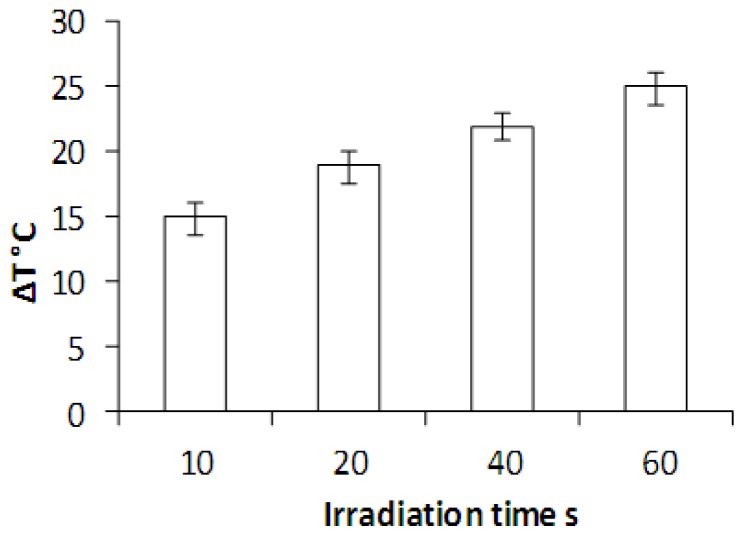
Temperature change in HNPs (50 ug mL^−1^) suspended in an agar phantom exposed to laser irradiation using a Q-switch Nd:Yag laser at 10 pulses s^−1^. All samples equilibrated to room temperature before beginning. Control sample consisted of 2% agar with no nanoparticles.

**Figure 9:**
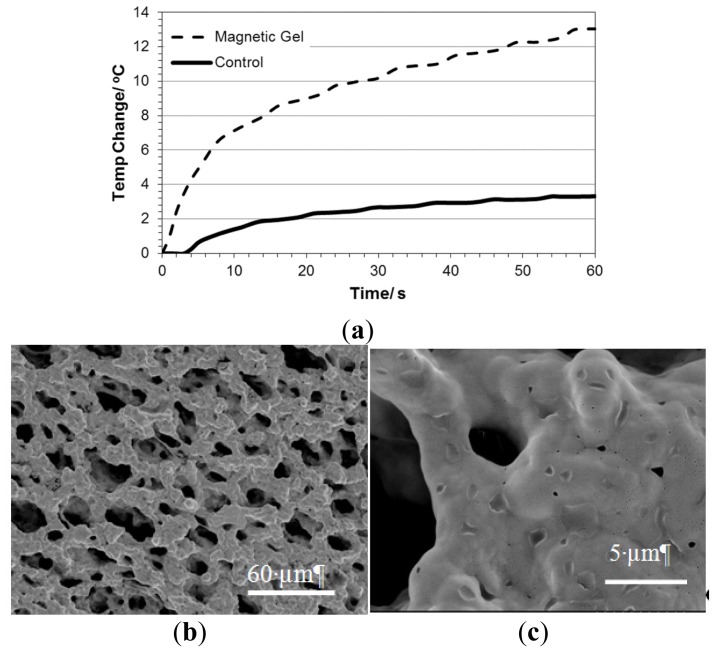
Physical properties of pNiPAM-HNP composite. (**a**) Temperature change in pNiPAM-HNP composite exposed to laser irradiation using a Q-switch Nd:Yag laser at 10 pulses s^−1^. FESEM images of composite (**b**,**c**).

Laser irradiation was carried out of HNP-PEG suspended in an agar (2%) phantom in 3.5 mm discs. The temperature increase was recorded at both the laser beam focus and in the surrounding sample to determine whether the heating was localized or whether bulk heating occurred (measured using thermocouples). Here, thermal increase was only experienced inside the laser beam diameter (7 mm) and no bulk heating was observed. Figure 8 shows the temperature change occurring when the HNPs were irradiated over 10, 20, 30, 40 and 60 s. The data suggests that the HNPs experienced an irradiation time dependent heating pattern, whereby, after 60 s the greatest temperature change of 25 °C was observed. This large increase in temperature over a short laser exposure time holds great potential for thermal manipulation of biomaterials such as scaffolds. Very minor thermal deviations were observed in the pNiPAM control, Figure 9, indicating the localization of heating effect to the nanoparticles.

In this study we used a nanosecond (Q-switched) pulsed laser fixed at 532 nm. As earlier discussed, a laser of wavelength 720 nm would be more appropriate for application due to the peak plasmon resonance observed after PEGylation, however, the peak absorbance on the UV-Vis spectra (Figure 3) was relatively broad and so heating effects were expected at 532 nm. Hence, these results may be greatly improved upon application of laser irradiation closer to the plasmon resonance wavelength.

### 2.5. Studies on pNiPAM-HNP Composites

**Figure 10:**
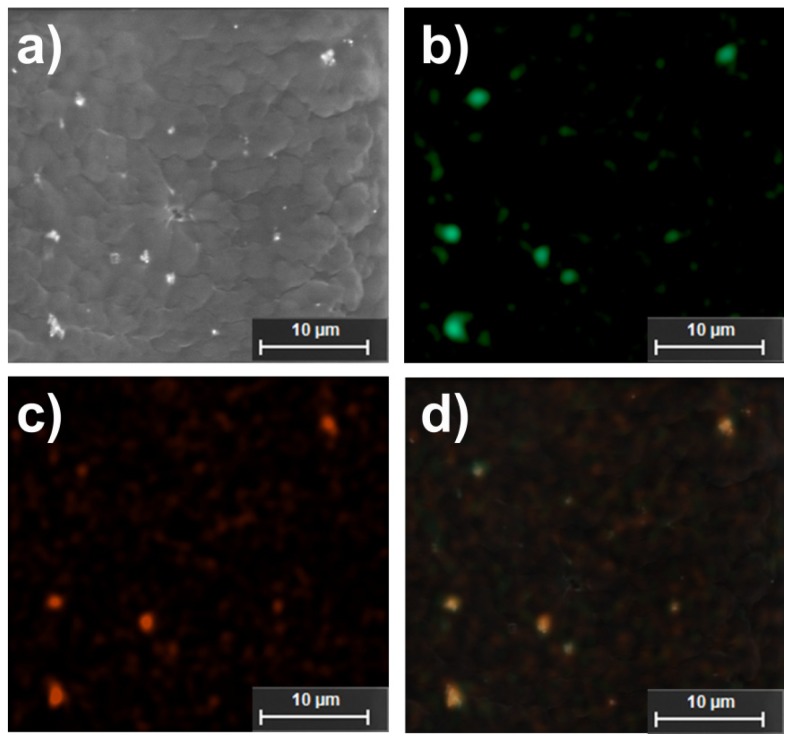
Electron microscopy and related EDX analysis indicating nanoparticle-polymer hybrid scaffolds. (**a**) SEM image; (**b**) iron; (**c**) gold and (**d**) overlaid iron and gold EDXA maps.

HNPs were successfully incorporated into the intrinsic structure of the scaffolds. This was evident from a color change in the bulk scaffold from a translucent pale white to a grey. Field Emission Scanning Electron Microscopy (FESEM) showed the matrix structure formed (Figure 9a). At increased magnification small nano-sized pores where evident which possibly were due to the HNP penetration into the intrinsic structure upon fabrication (Figure 9c). SEM EDX confirms the presence of HNPs inside the pNiPAM matrix (Figure 10). The pNiPAM-HNP composites were exposed to laser irradiation over 60 s and the temperature change measured using thin wire thermocouples. Figure 9a shows the temperature increase for the nanoparticle containing scaffold at 13 °C over the exposure period. Control scaffolds not containing nanoparticles were observed to heat minimally ~3.5 °C. Only those scaffolds hosting hybrid nanoparticles were distorted by the temperature changes induced by the irradiation, leading to expulsion of previously entrapped solvent out of the polymer network. This ability to manipulate scaffold integrity is thought to be beneficial in controlled drug delivery. Encapsulation of drug into the scaffold pores which could be released upon external stimulus could revolutionize controlled drug delivery. In addition, the inherent magnetism of the HNP core is suitable for MRI guidance; amalgamation of these two properties holds great potential for image guided prolonged drug delivery.

### 2.6 Stimulated Release of Model Compound

**Figure 11:**
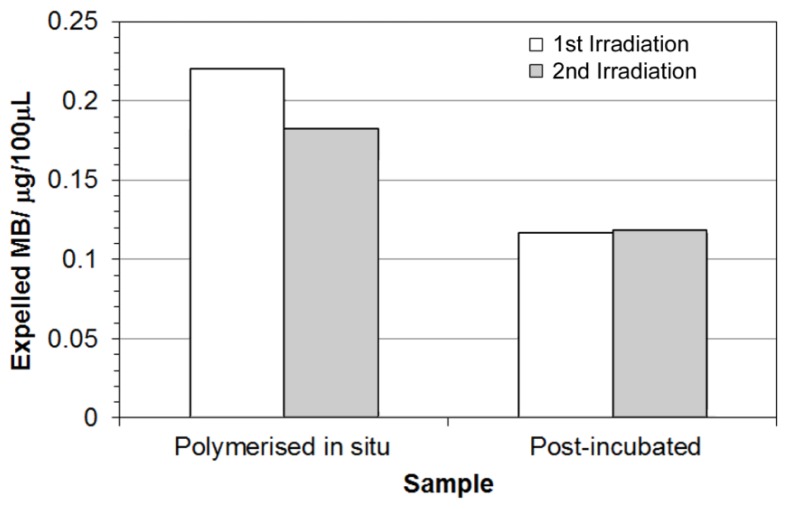
Expelled amount of methylene blue during laser irradiation of hybrid scaffolds.

pNiPAM-HNP composites were prepared containing methylene blue (MB) dye as a model drug, chosen for its intense absorption in the UV/Vis range allowing visualization during expulsion from the scaffold after thermal deformation. Gels were prepared either polymerized with dye *in situ* or post incubated in dye solution. All gels were rinsed thoroughly prior to laser irradiation to remove any surface dye, with one of the scaffolds formed with MB in the mix also left rinsing in dH_2_O for 2 days prior to experimentation. Upon laser irradiation samples were again found to deform, expelling solvent carrying MB out of the polymer network. This solution was harvested and assessed for quantification of MB using UV-Vis spectroscopy. Figure 11 shows that less dye was released in the post-incubated scaffolds, however this is probably due to a reduced quantity of dye being incorporated into the pore network through diffusion controlled ingress. Pre-loaded scaffolds have a higher amount of dye within the structure, so upon deformation larger amounts can be released giving rise to higher levels of expelled dye. The same samples were irradiated twice, with a short time in-between to remove any expelled solvent volume. Where pre-loaded samples showed a slight decrease in the amount of dye released during the second irradiation/deformation, similar levels were observed for scaffolds post-incubated in dye solution. This again may be due to the locality of the dye near to the surface of the gel. These results show clearly the ability to remotely control switching of the hybrid scaffold materials to cause thermal deformation, resulting in release of a model drug compound.

For scaffolds pre-loaded with the dye during polymerization, more dye was released during the first irradiation compared to the second.

## 3. Experimental Section

All materials were purchased from Sigma-Aldrich (Dorset, UK) unless otherwise stated.

### 3.1. Scaffold Formation

The pNiPAM scaffolds were analyzed using Fourier Transform Infrared Spectroscopy (FTIR) in order to characterize bonding structure in the polymer. pNiPAM gels were measured using a single bounce germanium attenuated total reflectance attachment (Nicolette IS50, Thermo-Fisher, Loughborough, UK). The samples were scanned taking an average of 128 spectra. Scaffolds were super critically dried after methanolic exchange, and imaged using scanning electron microscopy (SEM) and energy dispersive X-ray analysis (EDXA).

### 3.2. Synthesis of HNPs

Iron oxide cores were synthesized using a previously reported precipitation reaction [10,36,37]. Briefly, sodium hydroxide (NaOH, 1.030 g) and potassium nitrate (KNO_3_, 1.820 g) in deionized water (H_2_O, 180 mL) was refluxed at 90 °C under nitrogen (N_2_) for 1 h. Iron (III) sulphate heptahydrate (Fe_3_O_4_.7H_2_O, 3.89 g) in sulphuric acid (H_2_SO_4_, 20 mL) was added to the reaction and heated for 24 h at 90 °C. The black precipitate was cooled on ice and washed in H_2_O (six times). The magnetic particles formed were easily separated from solution using a high powered magnet and resuspended in water. A poly(ethylenimine) (PEI) intermediate was added via probe sonication of 5 mL magnetic particles with 5 mg mL^−1^ polymer in deionized water. Gold seeds were electrostatically attached to the polymer followed by reduction of HAuCl_4_ onto the particle surface forming a complete shell [38]. Hybrid nanoparticles were surface functionalized with cysteamine, allyl methyl sulphide, poly(ethylene glycol) and mercaptodecane via dative covalent bonding between the HNP (5 mg mL^−1^) and the SH groups in the polymers (5 mg mL^−1^). The final particles were washed extensively with deionized water to ensure excess polymer was removed before re-suspending in 5 mL deionized water.

### 3.3. Characterization of HNPs

Metal quantification was determined using inductively coupled plasma-optical emission spectroscopy (ICP-OES, Optima 7000V DV, PerkinElmer, Wokingham, UK). An acid digestion was carried out in concentrated nitric acid (HNO_3_, 1:5 sample:acid). The samples were diluted with deionized water prior to analysis. A calibration was run using iron and gold standard solutions 0.5–5 mg mL^−1^ (*R* = 0.9999). The concentration of HNPs used for all experiments indicates the concentration of Fe. Peak absorbance of HNPs in deionized water was measured using a Varian UV-Vis Cory 50 Bio spectrometer (Agilent, Wokingham, UK). Samples were analyzed in quartz cuvettes, absorbance scans were carried out between 200–800 nm. Polymer coating was determined using FTIR analysis as previously described. Hydrodynamic diameters and polydispersity index were estimated using photon correlation spectroscopy (PCS) (Zetasizer Nano-ZS, Malvern Instruments, Malvern, UK). All measurements were carried out at 25 °C (*n* = 3) and an average value determined. Zeta potential measurements were carried out to determine surface charge using the same instrument. Transmission electron microscopy (TEM) was used to visualize the particles. Samples were pipetted onto formvar coated copper grids (2 μL) and dried under a heat lamp for 4 h. The grids were directly imaged using a JEOL JEM-1230 microscope with ANAlysis software (JEOL, Japan). Magnetic characterisation was carried out in a Quantum Design MPMS-XL SQUID magnetometer as previously described [10]. Briefly, zero-field-cooled warming (ZFCW) and field-cooled-warming (FCW) and cooling (FCC) curves were measured between 10 and 280 K, in a field *H* = 8 kA/m. Magnetization *versus* applied field hysteresis loops were collected at 10 and 250 K in applied fields up to 4 MA/m.

### 3.4. Biological Characterization of HNPs

All tests were carried out on mouse osteoblast (7F2 cells). Cells were cultured in Alpha minimum essential media (MEM) (LifeTechnologies, UK) supplemented with 1 mM L-glutamine, 1 mM sodium pyruvate, 10% fetal bovine serum (LifeTechnologies, Paisley, UK) and 1% penicillin streptomycin.

Cell viability was estimated using trypan blue exclusion. Briefly, cells were seeded in 6-well plates (50,000/well) and cultured for 24 h. Media was removed and cells were incubated with polymer coated HNPs (6.25–100 μg mL^−1^) diluted in fresh media for 24 and 72 h. The cells were washed three times with phosphate buffered saline (PBS) before trypsinization. Cells were resuspended in 1 mL fresh media. Cell suspension (50 μL) was mixed with 50 μL trypan blue dye (LifeTechnologies, Paisley, UK) in an Eppendorf tube and viable cells were counted on an automated cell counter (Countess™, Life Technologies, Paisley, UK). Cellular uptake was determined using cells seeded in 6-well plates (50,000/well) incubated with HNP-PEG at final concentrations of 25 μg mL^−1^ over 1,4 and 24 h. The medium was removed, and the cells washed three times with PBS, trypsinized and resuspended in 1 mL medium. The cell number was counted using the Countess™, and cells were placed in Eppendorf tubes (1 × 10^6^ cells/tube). The cell suspensions were centrifuged at 800 rpm for 10 min, and the supernatant was removed. Concentrated hydrochloric acid (100 μL) was added to the tubes and these were incubated at 90 °C for 0.5 h. After cooling to room temperature, samples were centrifuged at 1500 rpm for 15 min. The supernatant was diluted with deionized water and run on an ICP instrument (Optima 7000V DV, PerkinElmer, Wokingham, UK). A calibration was carried out using iron standard solutions of 0.5 to 5 μg mL^−1^ (*R* = 0.9999).

The effect of the HNP-PEG on cell membrane integrity was estimated via measurement of lactate dehydrogenase (LDH) leakage from cells. Briefly, the cells were seeded in 96-well plates (15,000/well) and incubated with HNP-PEG (0–100 ug mL^−1^) for up to 72 h. After incubation, 2 μL of lysis buffer was added to the positive control wells, and the plate was centrifuged at 1000 rpm for 5 min at 37 °C. The supernatant (50 μL) was removed from individual wells and placed into a clean plate, 50 μL of a membrane integrity assay reagent was added to the wells. The plates were incubated for 10 min at 37 °C protected from light. Stop reagent (25 μL) was then added to the wells, and the fluorescence of the samples was measured at 560 nm (excitation) and 590 nm (emission) on a microplate reader (Pro200, Tecan, Reading, UK). The percentage of cytotoxicity with respect to the positive control wells was calculated, whereby the lysed cells were assumed to have 100% LDH release.

### 3.5. Laser Irradiation of HNPs in Agar Phantom

HNPs (50 ug mL^−1^) were dispersed into a 2% agar phantom in 35mm petri dishes (Greiner, UK). The samples were exposed to laser irradiation at 532 nm using a Q-switched Nd:YAG laser (10 ns pulses, 10 pulses/s). The beam was collimated in a 7 mm diameter and passed through the gel center. The temperature change was monitored using 0.076 mm diameter PFA coated T-type thermocouples (Omega, UK). One thermocouple was positioned at the center of the gel with a second placed at the edge of the gel as a control. All measurements were carried out at room temperature, 25 °C. Sample irradiation was carried out over 1 min. The temperature change in a control sample (2% agar) was measured in the absence of nanoparticles.

### 3.6. Incorporation of HNPs into pNiPAM Scaffolds

Samples were cut into ~1 mm thick sections using a razor blade, super critically dried and mounted onto aluminium stubs with carbon tape ready for SEM/EDXA using a bench top Hitachi TM3000 system (5 kV). For this samples were uncoated.

Field Emission Scanning Electron Microscope (FESEM, Hitachi, Japan) was conducted in a similar fashion using a Hitachi S4500 (Hitachi, Japan), coating samples with a flash of gold prior to analysis.

### 3.7 Laser Irradiation of pNiPAM-HNP Scaffolds

pNiPAM-HNP scaffolds were prepared into 1.5 mL Eppendorf tubes. The tubes were clamped horizontally and irradiated with a Q-switched Nd:YAG laser (10 ns pulses, 10 pulses/s) fixed at 532 nm. The beam was collimated in a 7 mm diameter and passed through the gel center. The temperature change was monitored using 0.076 mm diameter PFA coated T-type thermocouples (Omega, Manchester, UK). One thermocouple was positioned at the center of the gel. All measurements were carried out at room temperature, 25 °C. Sample irradiation was carried out over 1 min. The temperature change in a control sample (pNiPAM) was measured in the absence of nanoparticles.

### 3.8. Model Pharmaceutical Release

Scaffolds were prepared as previously described, with methylene blue being added into the polymer either upon formation (1 mg mL^−1^ in water solvent) or being soaked in via solvent replacement after polymerization (monolith immersed in 1 mg mL^−1^ MB aqueous solution for 2 days). To remove any surface dye which was not entrapped within the polymer scaffold, all materials were rinsed thoroughly prior to laser irradiation. Aliquots of 100 μL were taken immediately after irradiation, the polymer rinsed and retuned for repeated cycle of thermal deformation. Samples were analyzed on a Tecan M200 Pro (Tecan, UK) plate reader. Samples containing no dye were used as controls.

## 4. Conclusions

We have shown that PEGylated hybrid nanoparticles give rise to the lowest toxicity when cultured with 7F2 osteoclast cells. Laser irradiation of the particles in agar and pNiPAM-HNP composites caused localized get heating within the materials. Temperature changes applied both environmentally and localized by laser irradiation caused structural changes in pNiPAM scaffolds. Further work is on-going to investigate these systems for drug incorporation and controlled release.

## Figures and Tables

**Table 1:** Poly(*N*-isopropylacrylamide) (pNiPAM) gel samples investigated using various polymer gel compositions.

Constiutent component	Required amount for 1 mL solvent			
	0.7 M (A)	0.5M (B)	0.3M (C)	0.1M (D)
Water	1 mL	1 mL	1 mL	1 mL
NiPAM	0.0795 g	0.0568 g	0.03408 g	0.01136 g
MBA	1.32 mg	0.943 mg	0.5658 mg	0.1896 mg
APS	0.01 mg	0.01 mg	0.01 mg	0.01 mg
TMEDA	4 μL	4 μL	4 μL	4 μL

**Table 2:** Hybrid nanoparticle characterization using photon correlation spectroscopy, zeta potential measurement and concentration measurements using inductively coupled plasma–optical emission spectroscopy (ICP-OES).

Particle	Metal content analysis ug mL^−1^		Size nm (±SD)	PDI (±SD)	Zeta potential mV (±SD)
				
	Fe	Au			
Fe_3_O_4_	7000	-	2250 (125)	0.540 (0.125)	−16.5 (1)
Fe_3_O_4-_PEI	1920	-	270 (11)	0.125 (0.004)	+47.4 (3)
Fe_3_O_4-_PEI-Au_SEED_	1025	45	190 (9)	0.254 (0.001)	+27.5 (1)
Fe_3_O_4_-PEI-Au_COAT_ (HNP)	990	370	115(5)	0.258 (0.011)	+10.5 (0)
HNP-Cysteamine	868	323	413 (13)	0.679 (0.082)	+30.4 (1)
HNP-allyl methyl sulfide	885	385	392 (59)	0.738 (0.147)	+39.1 (4)
HNP-PEG	898	377	141.9 (4)	0.321 (0.054)	+20.5 (1)
HNP-mercaptodecane	924	370	442 (21)	0.921 (0.137)	+23.6 (2)
